# Financing Costs and Health Effects of Air Pollution in the Tri-City Agglomeration

**DOI:** 10.3389/fpubh.2022.831312

**Published:** 2022-03-04

**Authors:** Piotr O. Czechowski, Konstancja Piksa, Piotr Da̧browiecki, Aneta I. Oniszczuk-Jastrząbek, Ernest Czermański, Tomasz Owczarek, Artur J. Badyda, Giuseppe T. Cirella

**Affiliations:** ^1^Faculty of Management and Quality Science, Gdynia Maritime University, Gdynia, Poland; ^2^Faculty of Management and Economics, Gdansk University of Technology, Gdansk, Poland; ^3^Clinic of Infectious Diseases and Allergology, Military Institute of Medicine, Warsaw, Poland; ^4^Faculty of Economics, University of Gdansk, Sopot, Poland; ^5^Department of Informatics and Environment Quality Research, Faculty of Building Services, Hydro- and Environmental Engineering, Warsaw University of Technology, Warsaw, Poland

**Keywords:** health costs, general regression analysis, respiratory infection, healthy cities, Poland

## Abstract

This paper examines the relationship between the presence of air pollution and incidence of selected respiratory diseases in the urban population of the Tri-City agglomeration. The study takes into consideration the specific character of the region, relating to coastal, and port-based shipping. Three research hypotheses formulated the study. General regression models were used to identify the health effects of air pollution and developed health costs were calculated in relation to the treatment of diseases. The findings have shown that air pollution and climatic conditions in the Tri-City aggravate the symptoms of bronchial asthma, while also increasing the number of cases of exacerbated chronic obstructive pulmonary disease and pneumonia. The evidence demonstrates the negative impact of shipping on the health condition of the inhabitants. The calculations have shown the extent of financial losses incurred in connection with the treatment of diseases found to have been caused by air pollution. The estimated health costs turned out to be significant for each of the examined diseases. The financial inefficiency of the Polish health care system has also been demonstrated. All the models have been identified for monthly data for the first time.

## Introduction

Numerous studies have shown that air pollution causes a wide range of health effects from respiratory irritation to systemic ailments such as hypertension and premature death as a result of stroke or sudden cardiac arrest (1–4). There is a growing body of research that demonstrates atmospheric pollutants may induce not only physical but also mental illness (5, 6). Findings also indicate that pollutants are among the potential causes of reduced intelligence and accelerated aging due to their instrumental role in the development of neurodegenerative diseases (4, 7–10). The primary area of concern for this paper is to examine the influence of air pollution on the incidence of specific respiratory diseases. The study will focus on the following three diseases: bronchial asthma exacerbation, chronic obstructive pulmonary disease (COPD), and pneumonia (Supplementary Note 1).

As it turns out, the spectrum of impact of air pollutants may be much wider than previously assumed. Specifically, the impact extends to the sphere of the economy (11). This is due to the fact that diseases caused by these substances generate broadly understood health costs (12–14). These are usually classified into the following three types of costs: direct, indirect, and social. The first type of cost, the so-called direct costs, relate to financial outlays required for the supply of health services, i.e., they include all costs arising from the utilization of resources needed to provide medical care to patients and resources supporting the process of medical treatment. They also include direct medical and non-medical costs. Direct medical costs include the costs of utilizing resources to provide the patient with health care—borne by the health care system. They include, among others, the costs of diagnostic tests and recovery treatment as well as medication, hospitalization, and medical counseling. On the other hand, direct non-medical costs makeup the costs of using resources that support the process of providing medical services by the health care sector. Direct non-medical costs, as opposed to direct medical costs, are not related to diagnostic procedures, treatment, or recovery and mainly include the costs of patient transport (15). They are expenses incurred by both the state and households (12). In the coming decade, air pollution is forecast to increase (16), as a consequence the number of cases and hospital admissions should increase and translate into higher health care related costs (17). Indirect costs include expenses related to the occurrence of a given disease—borne by both the patient and the economy (15, 18). Within that first aspect, the losses suffered by the patient relate to their absence from work or diminished productivity due to illness. This may be associated with a decline in household income and, consequently, a reduction in consumer activity (19, 20). Reduced productivity and reduced employee attendance also adversely affect the productive capacity of businesses, as the disease often affects people's ability to work. Absence from work or the reduced productivity of a sick employee, in turn, decreases output (21–23). The sum total of these effects, observed at the accumulative level of all enterprises, reflects the costs incurred by the entire economy as a result of disease, i.e., costs which represent a reduction in the gross domestic product (22). Lower income of individuals and corporate bodies translates into lower amounts raised in taxation and social security contributions. Moreover, the health detriment suffered due to the presence of pollutants in the air generates an increased payout of social benefits. In the longer term, it also burdens the economy (15, 24). In 2015, the exposure of Polish employees to particulate matter (PM_2.5_) contributed to the loss of 16 million working days. This resulted in an estimated cost of EUR 2.1 billion per annum. Occasionally, a disease caused by air pollution may result in premature death which can result in losses in the amount of potential goods and services that could be produced by the worker by the time they enter into retirement (15, 21, 23). Social costs include all disease-related costs that are borne by society (25, 26). These are somewhat difficult to calculate, as they particularly involve the non-measurable aspects of the disease. They are associated not only with pain, but also with opportunities of taking advantage of goods being lost due to illness-related limitations. This generates physical as well as mental discomfort, contributing to a lower quality of life (15).

Air pollutants can be released into the atmosphere by natural emitters, such as volcanic explosions and forest fires or man-made fires (27). The most important group of emitters (i.e., at least in Poland) include combustion processes in municipal and housing sources and road transport, while less prevalent but nevertheless significant include energy production and distribution as well as the production activity of industrial plants. Agriculture and the waste management sector are also important sources (1). In the case of coastal regions, sea shipping is of particular importance in the process of air degradation. It is estimated that ships emit 15% nitrogen dioxide (NO_2_), 3–7% sulfur dioxide (SO_2_), and 3–8% soot—globally (28). The threat is posed not only by ships calling at the seaport, but also those mooring at the quay. Furthermore, on-board power generators constantly emit fumes during cargo handling operations (29). This paper explores two main objectives. First, it identifies the health effects of air pollution in the Tri-City agglomeration by modeling its causes. Second, it focuses on structuring its financial valuation. Due to limited access to economic data, only direct health costs will be estimated. An additional goal is to determine whether operations relating to coastal and port-based shipping, i.e., the urban industrial vicinity of the studied area, may have an effect on the health of the Tri-City population (Supplementary Figure 1).

## Methods

### Research Hypotheses

The following three hypotheses have been formulated for the study.

*H1*: The air pollution and climatic conditions in the Tri-City contribute to the exacerbation of asthmatic symptoms, the number of COPD exacerbations, and pneumonia.

*H2*: Shipping activity adversely affects the health of the Tri-City population.

*H3*: The health effects of air pollution generate costs that are borne by people living in the city.

A pilot study has been undertaken to test the hypotheses. The conceptual plan of the study can be extended to research other urban and industrial agglomerations.

### Materials

**Figure 1:**
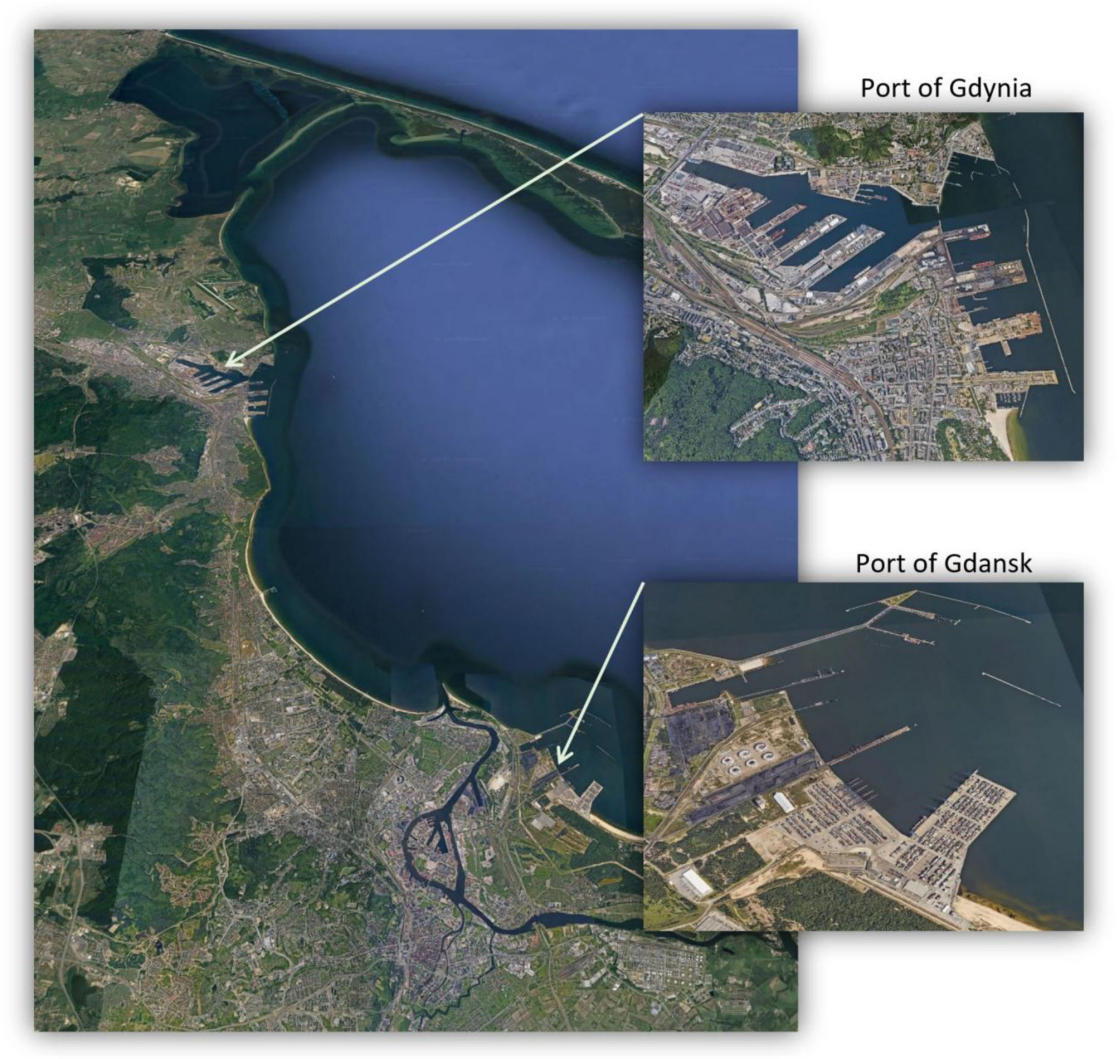
Tri-city agglomeration, consisting of two seaports. Source: Google Earth, 2021.

**Figure 2:**
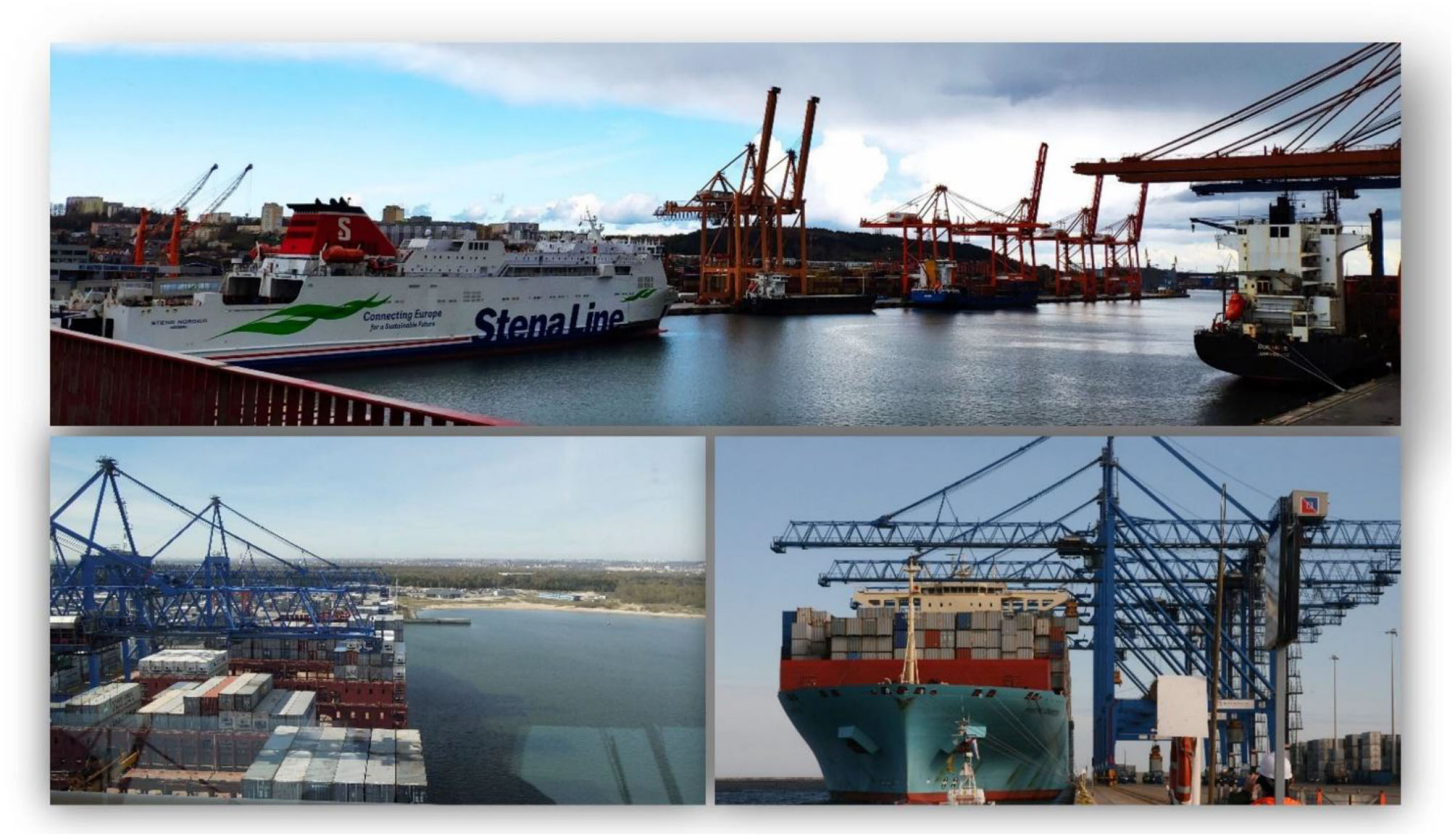
*(Top)* Port of Gdynia and *(bottom)* Port of Gdansk (top photograph taken by Aneta I. Oniszczuk-Jastrząbek on 25 April 2021; bottom photographs taken by Ernest Czermañski on 7 March 2018).

The study area of the Tri-City agglomeration consists of two seaports: Port of Gdansk and Port of Gdynia (Figures 1, 2). Various data sources have been used in the study. Health data relates to all health services provided by the National Health Fund (NFZ) in the period from 1 January 2010 to 31 December 2018 in the Tri-City area. These services were provided in connection with the occurrence of exacerbated symptoms of bronchial asthma, COPD, and pneumonia. Environmental data represents the measurements of air pollutant concentrations and weather factors supplied by reference devices averaged to the 1-h interval in the years 2010–2018 (i.e., taken on a daily basis and then interpolated to hourly intervals) (30). Atmospheric pollutants selected for preliminary testing included: carbon monoxide, carbon dioxide, nitric oxide, NO_2_, nitrogen oxide, ozone (O_3_), PM_10_, PM_2.5_, SO_2_, and benzo(a)pyrene. After substantive validation and problems with the quality of the input data, the following four atmospheric pollutants were used: NO_2_, O_3_, PM_10_, and PM_2.5_. Weather-related factors extended to include parameters such as air temperature, atmospheric pressure, wind speed and direction, air humidity, and rainfall total. Environmental data was obtained from the measuring stations of the Provincial Inspectorate for Environmental Protection located throughout the Tri-City area (28). The data includes the results of measurements taken by both land and sea winds. Data related to air pollution emissions generated by vessels were obtained from the Port of Gdansk. This data represents the total number of ship entries and exits recorded from 1 January 2010 to 6 November 2017 (31, 32). Due to difficulties in sourcing economic data, the required analytical material was obtained from the Military Institute of Medicine in Warsaw. The extrapolated data provided the hospitalization time and costs of provided health services with respect to diseases, whose incidence is related to the degree of air pollution. The obtained data reflects the costs that were incurred in each financial category related to: charging days, medical care, diagnostic procedures, and pharmacotherapy during hospitalization. The cash outlays were spent in the period from 1 January 2019 to 31 October 2019. The financial element of the analytical material was extended to include the refunded amounts allocated by the NFZ for the provision of specific medical services.

### Analytical Modeling

Before processing the data for the analytical modeling, the obtained health data and ship traffic were totaled and summed, and the environmental data was averaged. In effect, the obtained data was aggregated to monthly values. Next, the distribution of the studied dependent variables was verified by using the Kolmogorov-Smirnov test with the Lilliefors correction and the Shapiro-Wilk/Francia test with the Royston correction. Deviations from the normal distribution were found. Therefore, it was assumed that the studied distributions of the explained variables are similar to the normal distribution (29). Then followed the calculation of the -Ln MNW measure which is a measure of the distance of the distribution of each variable from the selected hypothetical distribution. This made it possible to assess the degree of similarity between the empirical and theoretical distributions. In each case, the closest distribution was the normal distribution, which in some cases required additional lossless transformations (i.e., logarithmization).

The identification of the health effects of air pollutants was based on constructed general regression models (GRM) using the general linear model (GLM) concept. With the help of GLM, it is possible to study complex experimental systems as well as interpret both qualitative and quantitative variables. This is of particular importance when analyzing data expressed in different measurement scales. An important advantage of GLM is its ability to describe curvilinear relations between variables. This allowed for the appropriate transformation of predictive factors as well as the utilization of substitution methods using the standardized variable *Z*. Such actions reduce the complexity of non-linear functions of the studied variables into a simpler form, thus making them linear. The GLM model estimates the parameters of the explanatory variables using the least squares method. In the event of a correlation occurring between the predictors, which is often the case with air pollutants, it is impossible to use this method due to the lack of the inverse of the X^T^X matrix. The GLM solves this problem by using the generalized inverse of the X^T^X matrix thus making it superior to other commonly used research methods such as multiple regression. Despite numerous advantages of GLM, when creating a GLM model, one should note its basic assumption relating to the normal distribution of the dependent variable (29).

A total of six GRM were built in two stages. First, they were built to integrate air pollutant emissions from both land and sea and, second, capture maritime emissions only. This division of labor was to emphasize the impact of maritime shipping activity on the incidence in the Tri-City population. In each model, the dependent variable was a selected disease. More precisely, it was the number of health services that were provided in connection with the occurrence of a given disease. Qualitative variables such as year (YEAR), seasonality (SS), quarters (QQ), months (MM), and quantitative variables accounted for on the independent variables—incorporating weather factors and air pollution concentrations. In order to identify the optimal GRM, the progressive stepwise regression method was used. This method allows selecting factors that have a statistically significant (i.e., *p* < 0.05) impact on the dependent variable. The mechanism of this method is as follows. First, an initial model is created. In step zero, it has no explanatory variables, but only the intercept. In each of the subsequent steps, separate models are built for all possible effects and verified (i.e., based on the Fisher-Snedecor test) in terms of statistical significance. If the value of the F-distribution calculated for a given effect exceeds the threshold value and, at the same time, is the highest among the others, this effect is entered into the model. The significance of the effects already included in the model is also tested at each step. In the event that it turns out to be statistically insignificant, they are removed from the created model. The step procedure is repeated until it is no longer possible to add or remove an effect. It will also be stopped if the maximum allowable number of steps has been used (33). To assess the quality of the created GRM, the corrected *R*^2^ was selected utilizing equation (1):


(1) $$\begin{array}{l} R^{2}=\;1-\frac{n-1}{n-k-1}\left(1-\frac{\sum_{vol=1}^{n}{\left({\hat{x}}_{vol}-\bar{x}\right)}^{2}}{\sum_{vol=1}^{n}{\left(x_{vol}-\bar{x}\right)}^{2}}\right) \end{array}$$

Where: *x*_*t*_= values of variable *x* at time or period *t*, ${\hat{x}}_{vol}$ = theoretical value of variable *x* at time or period *t*, *x* = mean value of variable *x* in a time series on *n* observations, *n* = number of observations, *k* = number of explanatory variables.

This is a measure that determines the extent to which the constructed statistical model explains the total variability of the studied phenomenon. The construction relies upon the determination of the sum of the squared deviations of the observed empirical values from the theoretical values established by the model. The sought sum ranges from zero to one. Negative values are possible in rare cases. When the value of this coefficient is zero, no relationship should be understood to exist between the dependent variable and the independent variables. On the other hand, the value of one equates to a perfect fit of the model to empirical data. Unlike an ordinary coefficient of determination, the corrected *R*^2^ will increase its value only in the event that the model is extended to include only those predictors that actually improve the degree of fit. This prevents us from making artificially inflated diagnoses about the quality of the model. This also indicates how well a model fits the data derived from a different sample drawn from the same population—expressed as a percentage (34, 35).

Health costs were calculated based on data aggregation summated for each individual economic and financial category relating to the process of treatment of diseases identified using the previously built GRM. For each disease, the total number of hospital admissions, hospitalization time, and treatment costs were calculated in four categories related to financing health services. The categories included charging day, medical care, diagnostic procedures, and medication. In the final phase, the financial result was calculated as the difference between the amounts refunded by the NFZ and the expenditure incurred in connection with the provision of specific medical services.

## Results

**Figure 3:**
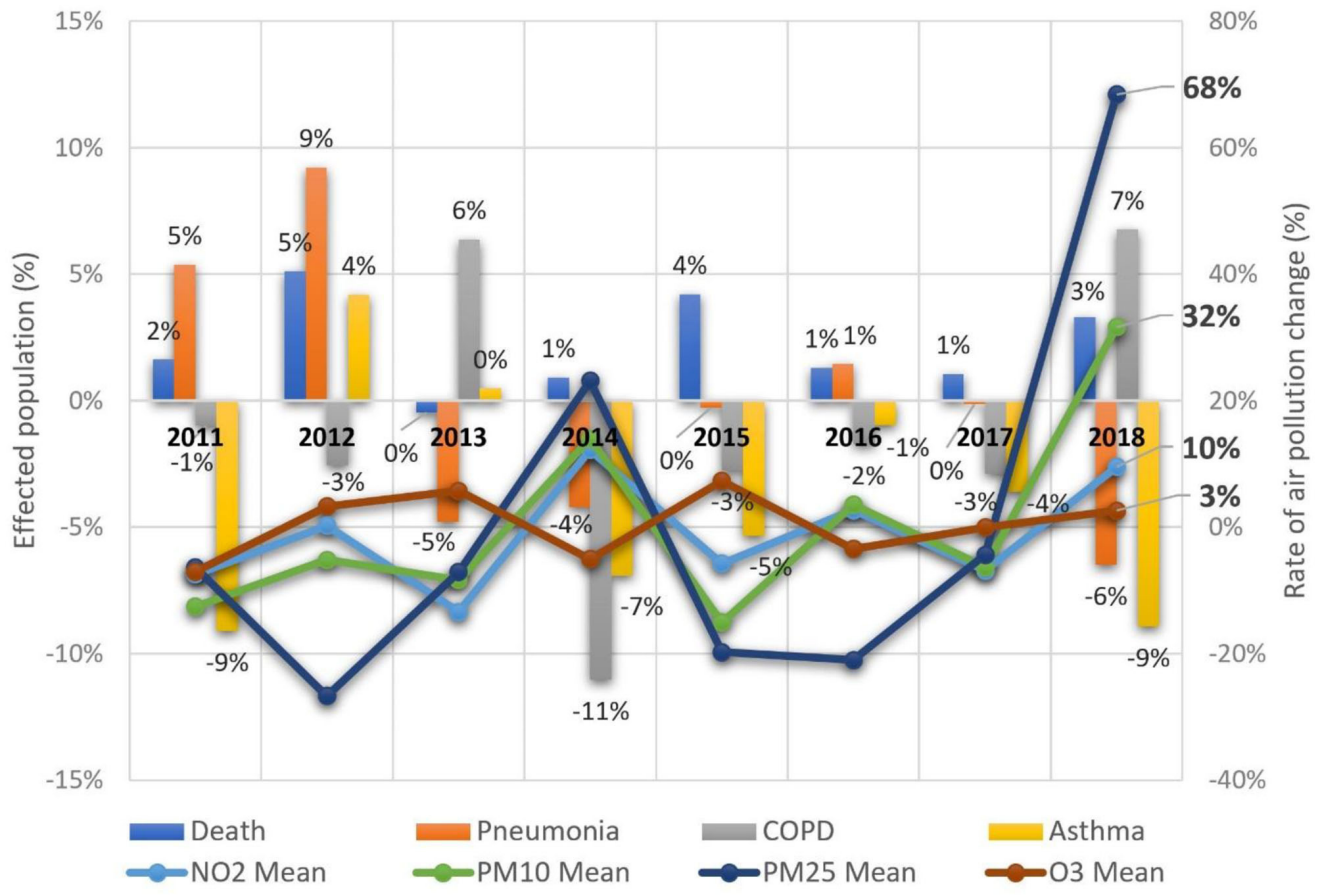
Dynamics of changes in the number of cases and deaths vs. air pollutants in the Tri-City, 2011–2018.

Factors that significantly contribute to health deterioration of the Tri-City population are illustrated in Table 1. Interactions between factors are ranked in order of correlation with the disease—based on the value of the F-statistics of the cause-and-effect models (see Supplementary Table 1 for the denotation of relating ICD10 codes and variables as well as Supplementary Data 1–5 for a detailed analyses of GRM used in the study). The timeline of the models ranged from 2010 to 2018. In the first model, both land and maritime emissions were included in the GRM. In terms of bronchial asthmatic symptoms, there was a 48.1% increase with the pollutants PM_10_ and PM_2.5_. This increase had a close correlation with seasonal weather temperature. COPD exacerbations accounted for a 43.1% increase with the pollutants NO_2_ and PM_2.5_. Temperature again emerged as an instrumental factor. In the case of pneumonia, a relatively high degree demonstrated a fit model in relation to the empirical data observed, i.e., 66.4%. A significant trigger factor for the aforementioned disease was PM_10_. A significant impact on the incidence was also attributed to the weather factors. In particular, the influence of air temperature, pressure, and wind speed. Figure 3 illustrates the dynamics of changes in the number of cases and deaths vs. air pollutants.

**Table 1:** GRM that consider interactions of air pollution and meteorological factors of the selected diseases and disease-related deaths in the Tri-City, 2010–2018.

**Disease**	**Bronchial asthma and status asthmaticus**	**Emphysema and other COPD**	**Pneumonia**	**Disease-related death**
ICD10 code	TRJ_J45 and TRJ_J46	TRJ_J43 and TRJ_J44	TRJ_J12 and TRJ_J18	TRJ_Death_all
Correlated *R*^2^	48.1%	43.1%	66.4%	50.0%
Factors	TRJ.PM10 ^*^ TRJ.TEMP	TRJ.TEMP	TRJ.PRES ^*^ TRJ.TEMP	TRJ.TEMP ^*^ TRJ.HUMID
	TRJ.PM25 ^*^ TRJ.TEMP	TRJ.PM25 ^*^ TRJ.TEMP	TRJ.PM10 ^*^ TRJ.WV	TRJ.PM25 ^*^ TRJ.WV
	YYYY	TRJ.NO2 ^*^ TRJ.TEMP		TRJ.TEMP ^*^ TRJ.PM10
				TRJ.PM25 ^*^ TRJ.RAIN

* *Interaction of factors*.

The results obtained using GRM that considered only maritime emissions are slightly different (Table 2). Bronchial asthmatic symptoms increased 34.1% due to the impact of emissions from the seaport. Among air pollutants, O_3_ and SO_2_ proved to be contributing factors for the aggravation of this disease. It is worth noting that SO_2_ also stood out, when full emission levels were reached, and may confirm it as an influencing pollutant. A significant role in shaping the studied phenomenon was also attributed to rainfall and air temperature. In terms of COPD, 64.9% made up the analyzed variability. Of particular importance is that both model types identified vessel activity as a significant predictor. Air temperature and rainfall also contributed to the number of COPD exacerbations. The maritime emissions model identified NO_2_ as a significant factor that contributed to the incidence of pneumonia. The corrected *R*^2^ was found to be 81.1% which highly correlates the analyzed variability. Moreover, O_3_ and PM_2.5_ were also attributed a significant role in the incidence of this disease. In terms of weather factors, only air temperature had a noticeable impact.

**Table 2:** GRM that consider only maritime emissions in terms of air pollution and meteorological factors of the selected diseases and disease-related deaths in the Tri-City, 2010–2018.

**Disease**	**Bronchial asthma and status asthmaticus**	**Emphysema and other COPD**	**Pneumonia**	**Disease-related death**
ICD10 code	TRJ_J45 and TRJ_J46	TRJ_J43 and TRJ_J44	TRJ_J12 and TRJ_J18	TRJ_Death_all
Correlated *R*^2^	34.1%	64.9%	81.1%	73.0%
Factors	YYYY ^*^ TRJ.RAIN.Wsea	QQ ^*^ TRJ.RAIN.Wsea	QQ ^*^ ShipNo	MM ^*^ TRJ.O3.Wsea
	TRJ.TEMP.Wsea ^*^ TRJ.RAIN.Wsea	YYYY	TRJ.NO2.Wsea ^*^ TRJ.O3.Wsea	YYYY ^*^ TRJ.PM10.Wsea
	TRJ.SO2.Wsea ^*^ TRJ.O3.Wsea	ShipNo	YYYY ^*^ QQ	TRJ.O3.Wsea ^*^ TRJ.PM25.Wsea
			YYYY ^*^ TRJ.PM25.Wsea	
			TRJ.TEMP.Wsea	

* *Interaction of factors*.

Both types of models identified shipping activity as a particularly important factor. A comparison of these models demonstrates a high level of chemical contaminants present in both ground level urban air and offshore landward air. This has been shown to worsen the symptoms of asthma (36–46) and increase the incidence of COPD (47–50) and pneumonia (51–54). Most of these health effects are also attributed to climatic conditions and shipping activities. Table 3 presents the results of the cost calculation related to the financing of selected health services. The data shows the total of expenses incurred by the Military Medical Institute from 1 January 2019 to 31 October 2019. As mentioned, the data concerns diseases for which pathogens were identified in the previous stage of the study.

**Table 3:** Financing costs of selected health effects of air pollution, 2010–2018.

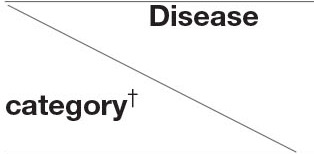	**Bronchial asthma exacerbation**	**COPD**	**Pneumonia**	**Total**
Total number of admissions	13	16	44	73
Total hospitalization time [days]	95	100	482	677
Total cost of charging days [PLN]	13,859	14,049	66,513	94,421
Total cost of medical care [PLN]	44,838	43,963	214,369	303,170
Total cost of diagnostics [PLN]	6,576	6,135	45,854	58,565
Total cost of medication [PLN]	2,814	1,572	31,747	36,133
Total treatment costs [PLN]	68,087	65,719	358,483	492,289
Total amount of refund [PLN]	45,973	35,891	110,098	191,962
Financial result [PLN]	−22,114	−29,828	−248,358	−300,327

*^†^USD 1 = PLN 4.1*.

The findings produced in this study demonstrate that the incidence of each disease caused by air pollution is associated with the need for considerable financial outlays. Particularly noteworthy is the negative financial result recorded for each of the studied diseases. This shows that the Polish health care system is characterized by financial inefficiency whose entire burden rests with centers providing medical services. The financial statement referenced in this paper contains an item that should command our particular attention, i.e., total hospitalization time incurred by all the studied diseases together—amounting to a total of 677 days. Based on this data, it can be noted that the group of hospitalized patients had working-age people in it. This suggests a link between the length of hospitalization with absenteeism from work. This could have contributed to a decline in the efficiency of enterprises, and, as a result, a reduction ensued in the value of goods and services produced.

## Discussion and Conclusions

The research produced findings pointing to a relationship between the presence of polluted air and the incidence of diseases within the urban population living in the Tri-City agglomeration. The study allowed for specific substances to be selected whose presence may likely be associated with certain inflammatory conditions in the respiratory tract. In the case of asthma, the substances are PM_10_ and PM_2.5_, in COPD it is NO_2_ and PM_2.5_, and in pneumonia PM_10_. The last finding confirms the results of studies carried out by Canadian scientists (5, 6). The impact of coastal and port-based shipping activities on the incidence of the studied respiratory system diseases was also proved. Shipping traffic turned out to be a significant contributing factor to the incidence of each of the studied diseases. The obtained results suggest that the limits of air pollutant emission established in Poland may be insufficient to protect human health. Therefore, their reduction should be considered based on additional research undertaken to bear out this hypothesis. Regulatory amendments should also apply to vessels. Adapting to potential legislative change may not be easy for every enterprise. Therefore, there are recommendations advising the introduction and improvement of the already existing market-oriented environmental policy tools. These policies should encompass a program of tradable permits for a certain amount of air pollutant emissions, appropriately increased emission fees or better-defined property rights. These are tools that not only enable companies to flexibly adapt to the applicable environmental regulations but also motivate them to reduce air pollutant emissions (55). The impact on the studied phenomenon has repeatedly revealed the influence of air pollutants which are largely emitted by road transport. These pollutants are particularly nitrogen oxides and PM. Therefore, there is a need for infrastructure that would divert traffic away from agglomerations. Air quality in the Tri-City would also be improved by the exclusion of diesel engine vehicles from traffic and the popularization of hybrid driven and electric cars (56–58). Another investment for the improvement of the current situation should aim to enlarge the size of eco-friendly fleets in public transport. The watercourses located in the Tri-City agglomeration also provide an opportunity for the development of inland water transport. Moreover, the estimation of the direct health costs showed the financial burden of polluted air. The financial outlays turned out to be significant for each of the diseases discussed. The seriousness of the problem is additionally aggravated by the fact that the study has revealed the financial inefficiency of the Polish health care system (59–61). This increases the burden of pollution-related diseases carried by medical service providers. In the longer term, it can be noticed that this is a problem not only of medical facilities, but also of public entities that finance their operation.

In this paper we achieved two important goals for the Tri-city agglomeration. First, it was identified that emitted air pollutants influence the health of the population. This not only negatively impacts the health of individuals but also burdens the agglomeration with trickle-on financial costs. These consequences, relating to the occurrence of disease caused by exposure to air pollution, in particular pneumonia, and exacerbations of the diseases such as bronchial asthma or COPD, inadvertently have economic knock-on effects that can render the provision of services less effective. Second, the GRM analyses indicated that air pollution and weather factors significantly influence the incidence of considered respiratory diseases and their exacerbations. Notably, these findings are similar to a number of other studies that looked at other air pollutants (6, 40, 52, 62, 63) outside the context of a seaport city. The applied models explain a large part of the variance in the occurrence of the diseases considered. The relatively high *R*^2^ coefficient, ranging from 43.1% for COPD and 48.1% for asthma to 66.4% for pneumonia, proves that a significant part of the variability in the occurrence of these diseases can be explained by changes in air quality and meteorological parameters. The noticeably greater part of the variance (i.e., with the exception of the bronchial asthma model), is explained by models that only account for maritime emissions. Regarding the economic aspect, the analyses elucidates health outcomes attributed to air pollution burdens the public health system via a number of factors. Serious losses by health care units have been reported based on the NFZ reimbursement scheme (29). These losses support the conclusion that the analyzed diseases are systematically underestimated. Therefore, it is necessary to conduct further research based on the presented cause-and-effect sequence in order to fully understand the mechanisms behind the impact of air pollutants.

## Data Availability Statement

The datasets presented in this study can be found in online repositories. The names of the repository/repositories and accession number(s) can be found in the article/Supplementary Material.

## Author Contributions

PC: conceptualization, data curation, formal analysis, investigation, modeling, statistical analysis, methodology, writing—original draft, and writing—review and editing. KP: modeling, statistical analysis, software, methodology, and writing—original draft. PD: medical analysis, validation, visualization, writing—original draft, and writing—review and editing. AO-J: economic analysis, methodology, visualization, writing—original draft, and writing—review and editing. EC: economic analysis, resources, supervision, writing—original draft, and writing—review and editing. TO: modeling, statistical analysis, methodology, data curation, formal analysis, and investigation. AB: data curation, data acquisition, environmental analysis, methodology, writing—original draft, and writing—review and editing. GC: methodology, visualization, and writing—review and editing. All authors read and approved the final manuscript.

## Conflict of Interest

The authors declare that the research was conducted in the absence of any commercial or financial relationships that could be construed as a potential conflict of interest.

## Publisher's Note

All claims expressed in this article are solely those of the authors and do not necessarily represent those of their affiliated organizations, or those of the publisher, the editors and the reviewers. Any product that may be evaluated in this article, or claim that may be made by its manufacturer, is not guaranteed or endorsed by the publisher.
