# Supplementary material for: Financing Costs and Health Effects of Air Pollution in the Tri-City Agglomeration
Source: Front Public Health. 2022 Mar 4;10:831312. doi: 10.3389/fpubh.2022.831312 (PMC8931043; doi:10.3389/fpubh.2022.831312)
Supplement: Supplementary file 1 [file Data_Sheet_1.PDF]

Supplementary Material

Financing Costs and Health Effects of Air Pollution in the Tri-City Agglomeration

Czechowski et al.

Supplementary Figure 1

Graphical Abstract

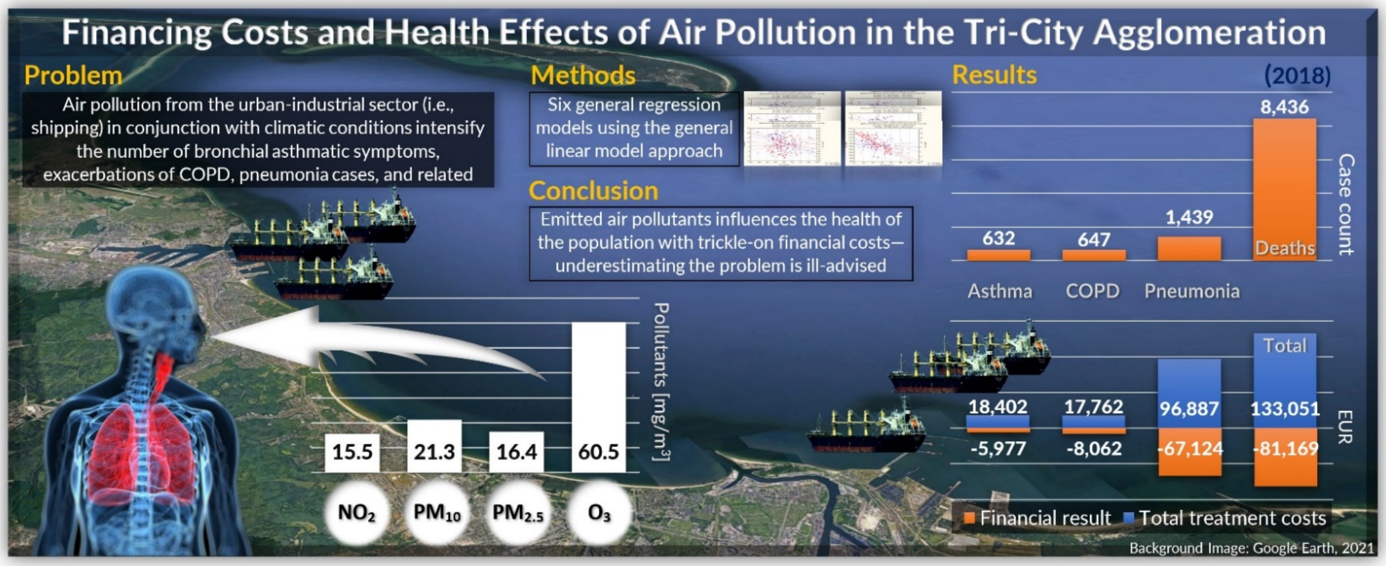

Supplementary Note 1

Asthma is a chronic disease of the respiratory tract that develops with the accompaniment of a chronic inflammatory process (1,2). It is most often the result of a strong immune system response to an allergen in the respiratory system. The allergen leads to inflammation, causing the airways to swell and constrict. A frequent effect of the allergen is also the contraction of the muscles surrounding the airways leading to bronchospasms and a feeling of breathlessness. These are very dangerous phenomena since they lead to breathing difficulties. Asthma affects people of all ages; however, it is mostly diagnosed in childhood (3,4). One of the exacerbating factors for asthmatic-based symptoms is air pollution from road traffic—in particular nitrogen dioxide (NO_2) (5,6) and particulate matter (PM) (7). Hospitals record a spike in the number of admissions related to asthma exacerbations on days when these substances occur at elevated concentrations (8)—including a noticeably increased need for drugs that alleviate symptoms (9,10). Jędrak et al. (10) found that a significant impact factor for the incidence of asthma and other respiratory diseases in children aged 7 to 16 corresponded to the distance from where they live to a busy road (i.e., the highest frequency of symptoms of this disease was observed in children living within 200 m versus the lowest more than 500 m).

COPD is a progressive lung disease that demonstrates itself in the feeling of exercise-related dyspnoea caused by chronic inflammation of the lungs, leading to decreased respiratory flow rate. Sometimes it leads to an inability to engage in everyday activities. Restricted lung ventilation is most often the result of damage to the alveolar walls. As a result, they lose their shape and flexibility, while also decreasing in number in what is known as emphysema (11–13).

Inflammation and swelling of the airways can also be an additional factor. This causes secretion of large amounts of mucus, resulting in difficulty breathing. COPD is usually diagnosed in middle age (i.e., after the age of 40) or in old age. Unlike asthma, changes to the airways caused by asthma are irreversible. Smoking is considered to be the most common cause of the disease (14,15). It is the reason for no less than 75% of diagnosed cases (16). Other risk factors are considered to include air pollutants (17)—primarily NO_2 and PM. It was investigated that non-smokers living in the capital city of Poland near busy roads are over four times more likely to develop bronchial obstruction than residents of rural areas (18). In other studies, also carried out in Poland, it was shown that non-smokers living less than 50 m from a busy road more often show signs of bronchial obstruction compared to people living more than 100 m, and especially more than 150 m (18–21). Nonetheless, the literature stresses the need for further research to confirm the proposed hypothesis. There is, however, evidence of a relationship between short-term exposure to the aforementioned pollutants and the aggravated symptoms of COPD in chronically-ill patients. It was established that an increase in $PM_{2.5}$ and PM_{10} by 10 µg/m^3 causes, within a relatively short timeframe, a hike in hospital admissions by 0.9% and 2.7%, respectively. Moreover, it was observed that at times of particularly high pollutant concentrations (i.e., especially during augmented levels of smog), medication is consumed in larger quantities due to an exacerbation of the disease symptoms (9,10).

It has been shown that exposure to polluted air also increases the incidence of infectious diseases of the respiratory system (10,22–24). This is confirmed by the results of the observation carried out in Utah, which stated that an increase in $PM_{2.5}$ dust concentration by 10 µm/m^3 contributed to an increase in the number of health services provided due to respiratory tract infections. In children under two years old, the increase was 15%, while in the second group of children aged 2-18, the same figure stood at 32%. Among adults, the reporting of respiratory tract infections increased by 19%. In 76.5% of children in the first age group, bronchiolitis was the main reason for the increased need for medical care (9). A particularly dangerous infection involving the lower respiratory tract is pneumonia. In 2017, it accounted for 15% of all deaths of children under the age of five. Various types of bacteria, viruses (i.e., including coronaviruses), and fungi contribute to pneumonia (25,26). The results of a Canadian study have also demonstrated the impact of air pollution on the incidence of the disease in the elderly. Long-term exposure to NO_2 and $PM_{2.5}$ at elevated concentrations were found to contribute to the incidence of community-acquired pneumonia in people over 65. It is believed that this may be due to damage to the epithelium lining the airways, possibly as a result of exposure to NO_2. As a consequence, there is a reduction in the function of mucociliary clearance as well as in the number of beneficial phagocytic cells. This allows pathogens to penetrate and grow in the alveoli (27).

Supplementary Data 1

Detailed analyses of GRM used in the study

Correlative breakdown of the study

Disease	*Bronchial asthma and status asthmaticus*	*Emphysema and other COPD*	*Pneumonia*	*Death*
**ICD10 code**	**J45_J46**	**J43-J44**	**J12_J18**	**TRJ_Death_all**
**Location**	TRJ	TRJ	TRJ	TRJ
**Correlated *R*²**	53.8%	63.2%	81.2%	80.3%
**df=n-1**	72	82	68	64
**MM**	2			2
**YYYY**	2	1	2	2
**SS**		1	1	2
**QQ**			3	
**TRJ.SO2**	1		1	1
**TRJ.NO**			1	
**TRJ.NO2**				
**TRJ.NOX**				
**TRJ.O3**		1		1
**TRJ.CO**		1		
**TRJ.CO2**	**1**			2
**TRJ.PM10**				1
**TRJ.PM25**	1			1
**TRJ.PRES**			1	
**TRJ.WV**		3	1	1
**TRJ.TEMP**	2	2	2	
**TRJ.HUMID**	1			
**TRJ.RAIN**	2	1	1	1
**ShipNo**	1	1	1	1
**TRJ.BaP**		2	2	2
Factors	TRJ.TEMP*TRJ.HUMID	TRJ.WV*ShipNo	QQ*ShipNo	YYYY*TRJ.PM10
	MM*TRJ.PM25	TRJ.RAIN*TRJ.BaP Mean	TRJ.NO*TRJ.BaP Mean	SS*ShipNo
	TRJ.RAIN*ShipNo	TRJ.WV*TRJ.TEMP	QQ*TRJ.BaP Mean	TRJ.O3*TRJ.WV
	YYYY*TRJ.RAIN	YYYY*TRJ.TEMP	TRJ.PRES*TRJ.TEMP	YYYY*SS
	TRJ.SO2*TRJ.TEMP	SS*TRJ.O3	SS*TRJ.WV	TRJ.CO2*TRJ.PM25
	TRJ.CO2	TRJ.CO*TRJ.WV	YYYY*TRJ.TEMP	MM*TRJ.BaP Mean
			YYYY*TRJ.RAIN	TRJ.SO2*TRJ.RAIN
			QQ*TRJ.SO2	TRJ.CO2*TRJ.BaP Mean

Bronchial asthma and status asthmaticus

J45_J46

Univariate results for each DV (##TRJ NFZ3 MM 2010_2018 in NFZ3 2010 MAIN v060 kor2021.stw)
Forward stepwise solution
Effective hypothesis decomposition

	Degr. of Freedom	TRJ_sum_J45_J46 SS	TRJ_sum_J45_J46 MS	TRJ_sum_J45_J46 F	TRJ_sum_J45_J46 p
Intercept	1	182898.457	182898.457	3424.99237	0
TRJ.PM10*TRJ.TEMP	1	617.84	617.84	11.57	0.00
TRJ.PM25*TRJ.TEMP	1	346.92	346.92	6.50	0.01
YYYY	8	2180.55	272.57	5.10	0.00
Error	97	5179.90946	53.4011284		
Total	107	9976.62963			

Dependent Variable	Multiple R	Multiple R2	Adjusted R2	SS Model	df Model	MS Model	SS Residual	df Residual	MS Residual	F	p
	Test of SS Whole Model vs. SS Residual (##TRJ NFZ3 MM 2010_2018 in NFZ3 2010 MAIN v060 kor2021.stw)										
TRJ_sum_J45_J46	0.6933943	0.480795655	0.427269433	4796.72017	10	479.67202	5179.9095	97	53.40	8.98	0.00

Mean plot and Pareto chart of t-Values

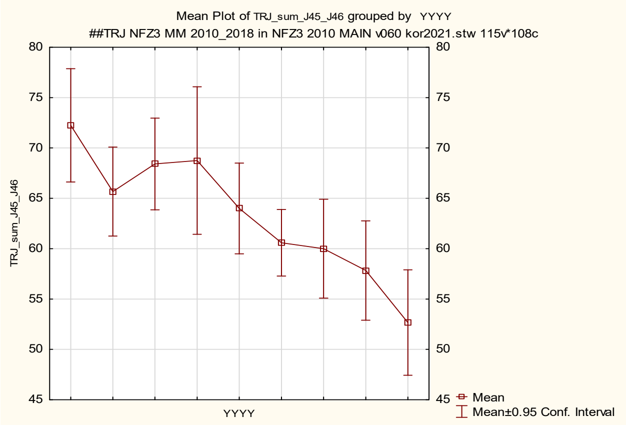

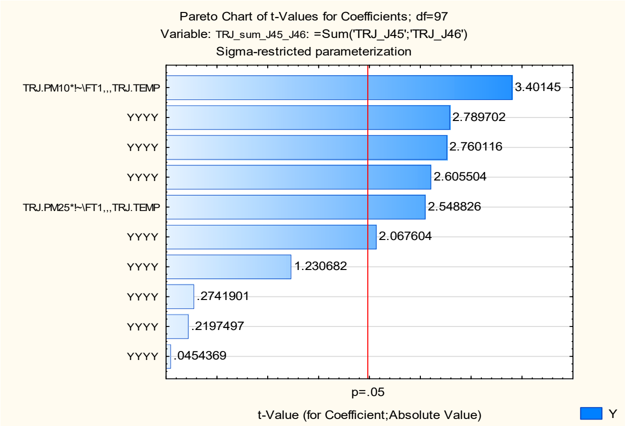

Supplementary Data 2

All groups and multiple variables

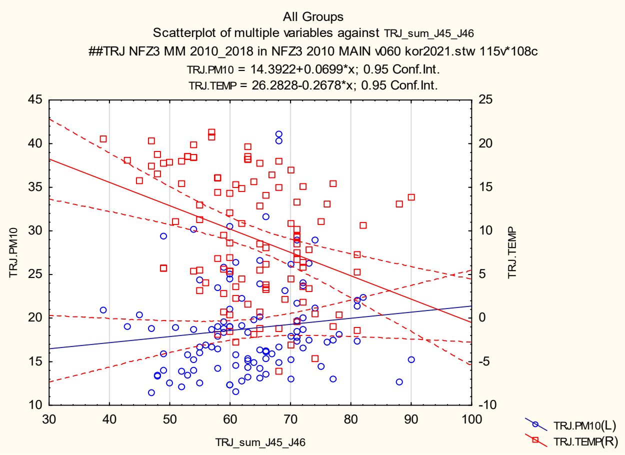

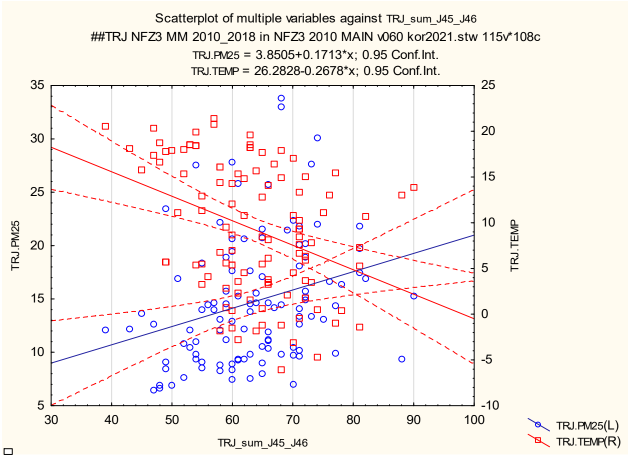

GRM PM10 and PM25
TEMP

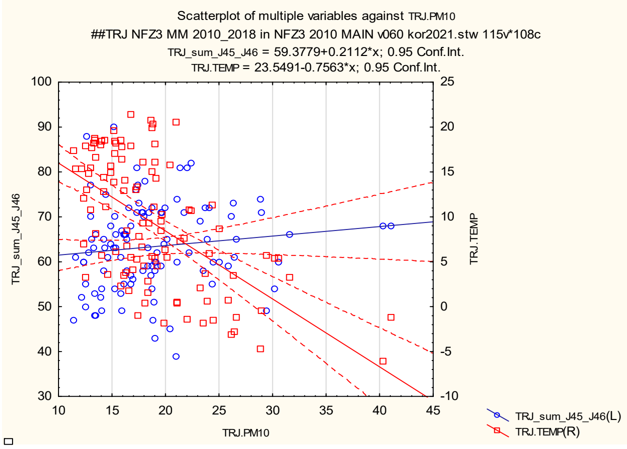

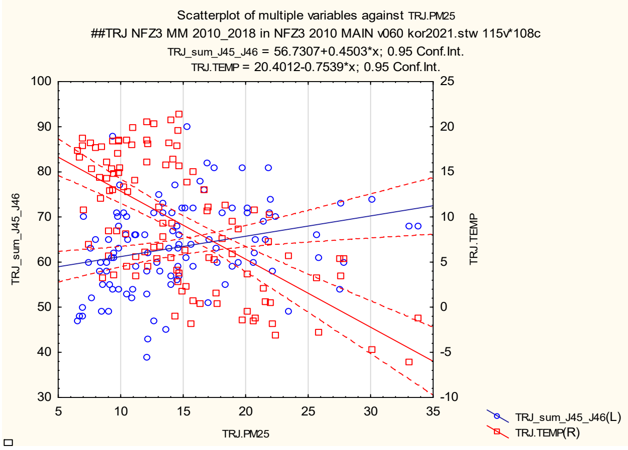

Emphysema and other COPD	Univariate results for each DV (##TRJ NFZ3 MM 2010_2018 in NFZ9_3 2010 MAIN v003.stw)
J43-J44	Forward stepwise solution
	Effective hypothesis decomposition

	Degr. of Freedom	TRJ_sum_J43_J44 SS	TRJ_sum_J43_J44 MS	TRJ_sum_J43_J44 F	TRJ_sum_J43_J44 p
Intercept	1	147921.693	147921.693	3570.68941	0
TRJ.TEMP	1	349.80	349.80	8.44	0.00
TRJ.PM25*TRJ.TEMP	1	666.17	666.17	16.08	0.00
TRJ.NO2*TRJ.TEMP	1	198.41	198.41	4.79	0.03
Error	104	4308.37139	41.426648		
Total	107	7578			

Test of SS Whole Model vs. SS Residual (##TRJ NFZ3 MM 2010_2018 in NFZ9_3 2010 MAIN v003.stw)

Dependent Variable	Multiple R	Multiple R2	Adjusted R2	SS Model	df Model	MS Model	SS Residual	df Residual	MS Residual	F	p
TRJ_sum_J43_J44	0.656858633	0.431463264	0.415063165	3269.62861	3	1089.8762	4308.3714	104	41.43	26.31	0.00

Scatterplot TRJ_sum against TRJ_TEMP

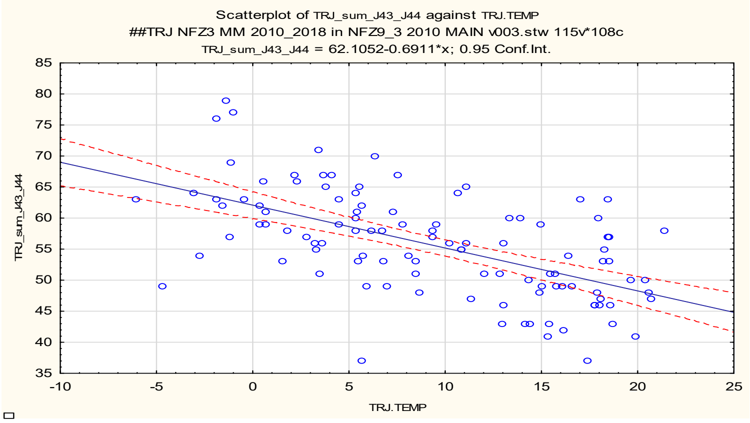

Supplementary Data 3

PM25 and TEMP

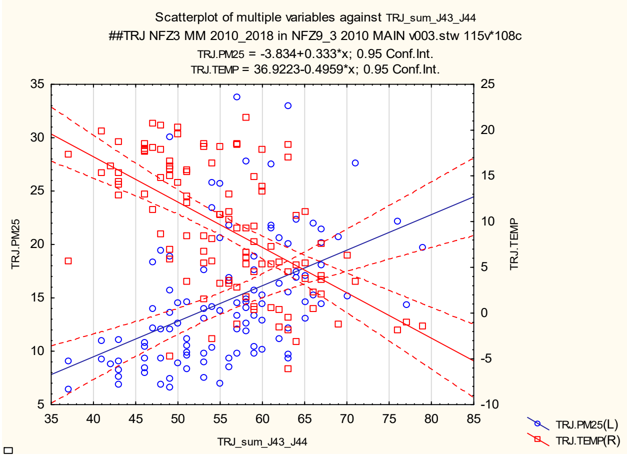

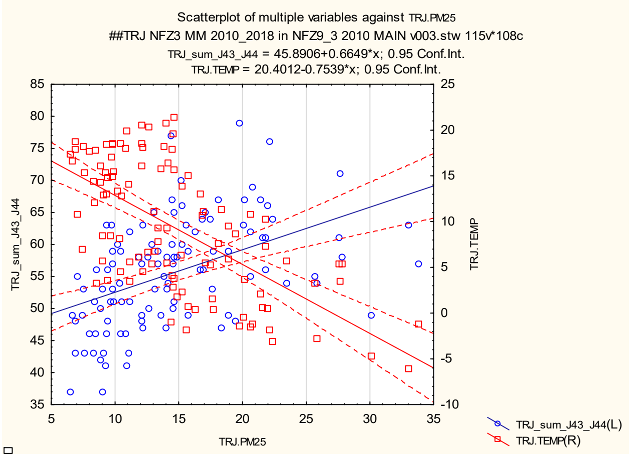

NO2 and TEMP

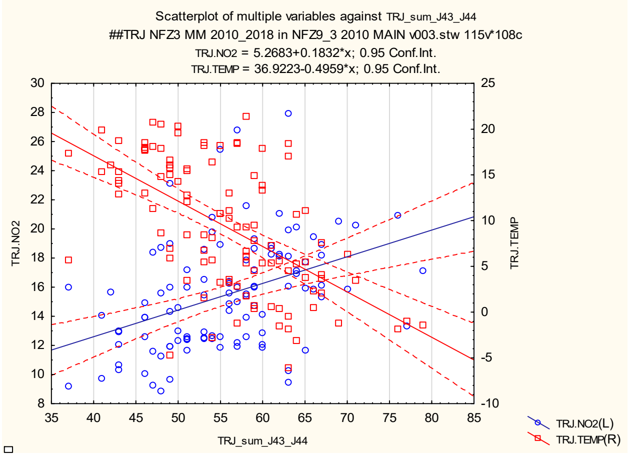

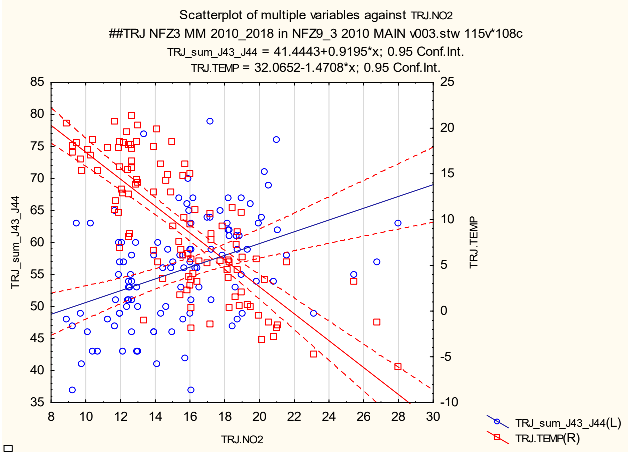

Pneumonia — **J12_J18**

Univariate results for each DV (##TRJ NFZ3 MM 2010_2018 in NFZ9_3 2010 MAIN v003.stw)
Sigma-restricted parameterization
Effective hypothesis decomposition

	Degr. of Freedom	TRJ_sum_J12_18 SS	TRJ_sum_J12_18 MS	TRJ_sum_J12_18 F	TRJ_sum_J12_18 p
Intercept	1	100721.86	100721.86	527.47	0.00
TRJ.PRES*TRJ.TEMP	1	23127.79	23127.79	121.12	0.00
TRJ.PM10*TRJ.WV	1	1450.06	1450.06	7.59	0.01
Error	105	20049.9487	190.951892		
Total	107	59592.1019			

Test of SS Whole Model vs. SS Residual (##TRJ NFZ3 MM 2010_2018 in NFZ9_3 2010 MAIN v003.stw)

Dependent Variable	Multiple R	Multiple R2	Adjusted R2	SS Model	df Model	MS Model	SS Residual	df Residual	MS Residual	F	p
TRJ_sum_J12_18	0.814583868	0.663546878	0.657138247	39542.1531	2	19771.08	20049.95	105.00	190.95	103.54	0.00

Scatterplot TRJ_sum against TRJ_TEMP

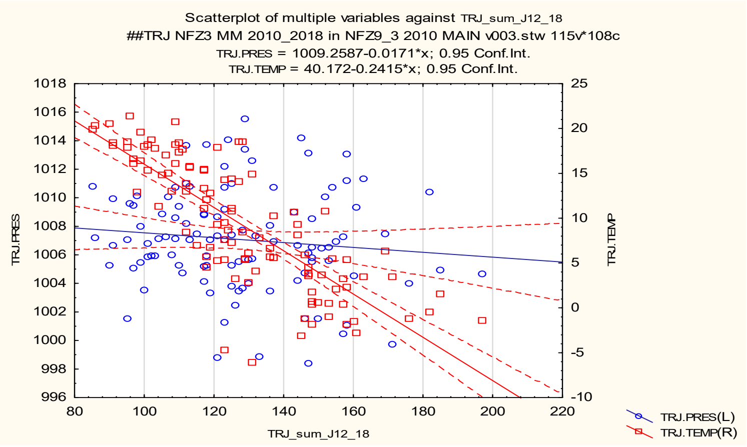

Supplementary Data 4

PM10 and TEMP

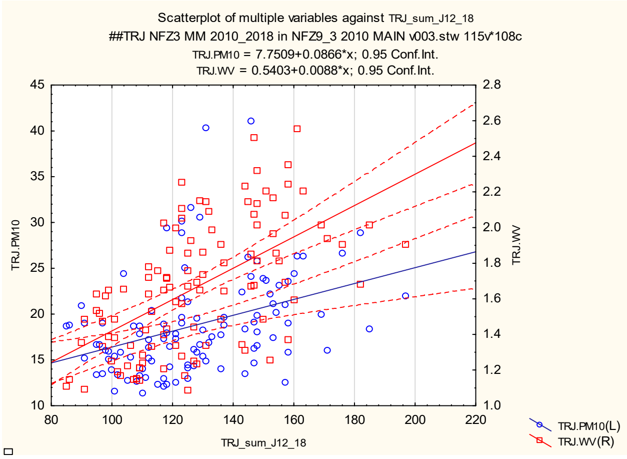

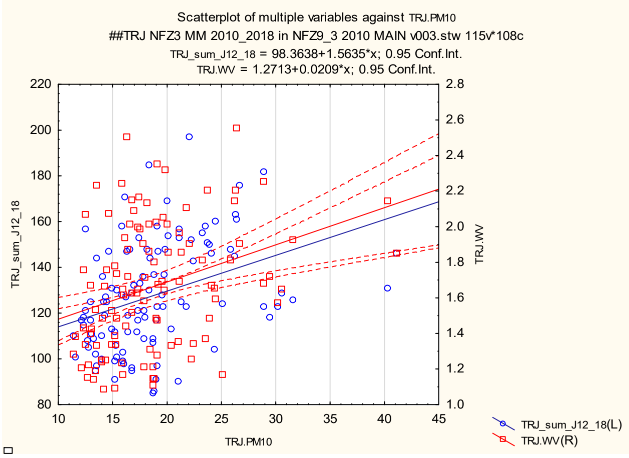

**Death**	Univariate Results for Each DV (##TRJ NFZ3 MM 2010_2018 in NFZ9_3 2010 MAIN v003.stw)				
**TRJ_Death_all**	Forward stepwise solution				
	Effective hypothesis decomposition				
		Degr. of Freedom	TRJ_death all SS	TRJ_death all MS	TRJ_death all F	TRJ_death all p
	Intercept	1	3233637.53	3233637.53	1530.41	0.00
	TRJ.TEMP*TRJ.HUMID	1	133091.24	133091.24	62.99	0.00
	TRJ.PM25*TRJ.WV	1	91118.70	91118.70	43.12	0.00
	TRJ.TEMP*TRJ.PM10	1	49960.39	49960.39	23.65	0.00
	TRJ.PM25*TRJ.RAINsum	1	21510.02	21510.02	10.18	0.00
	Error	103	217630.47	2112.92		
	Total	107	434935.21			

Dependent Variable — Test of SS Whole Model vs. SS Residual (##TRJ NFZ3 MM 2010_2018 in NFZ9_3 2010 MAIN v003.stw)

Dependent Variable	Multiple R	Multiple R2	Adjusted R2	SS Model	df Model	MS Model	SS Residual	df Residual	MS Residual	F	p
TRJ_death all	0.70684195	0.499625542	0.480193524	217304.742	4	54326.185	217630.47	103	2112.92	25.71	0.00

TRJ.TEMP*TRJ.HUMID

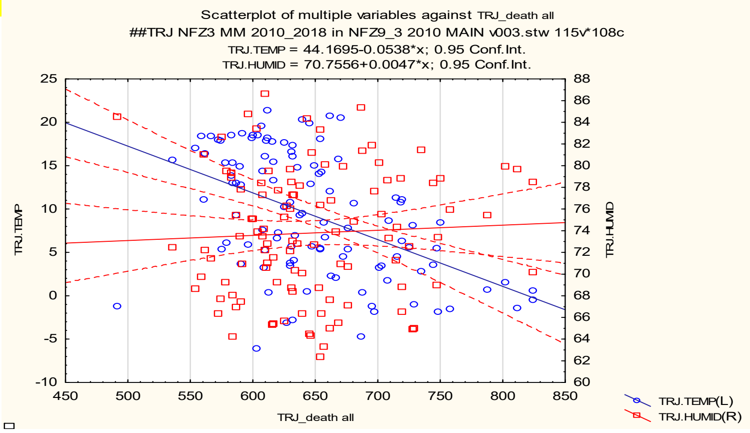

TRJ.PM25*TRJ.WV

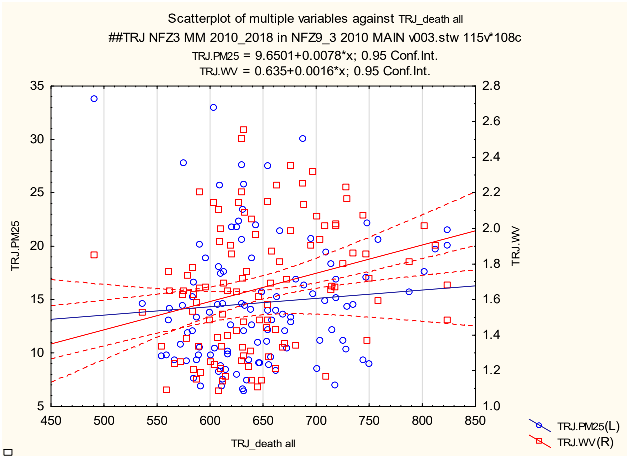

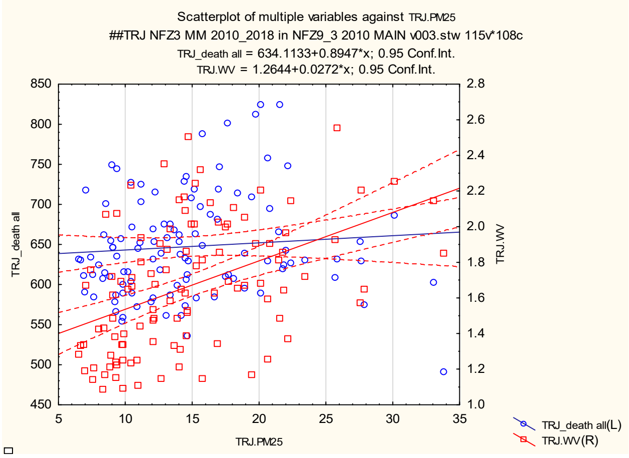

Supplementary Data 5

TRJ.TEMP*TRJ.PM10

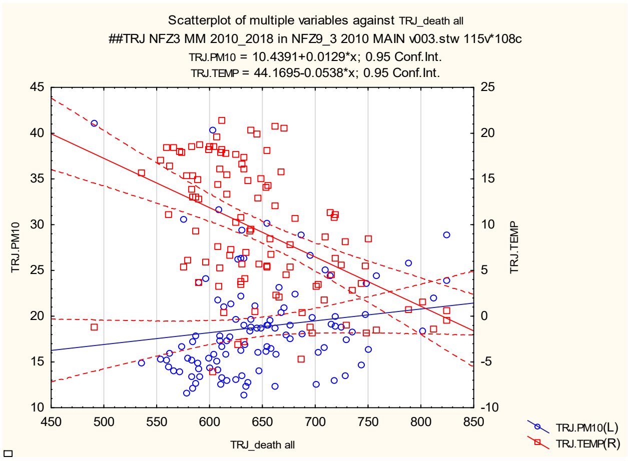

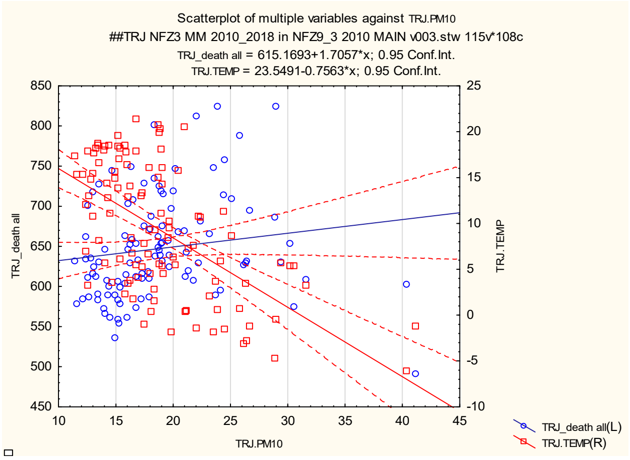

TRJ.PM25*TRJ.RAINsum

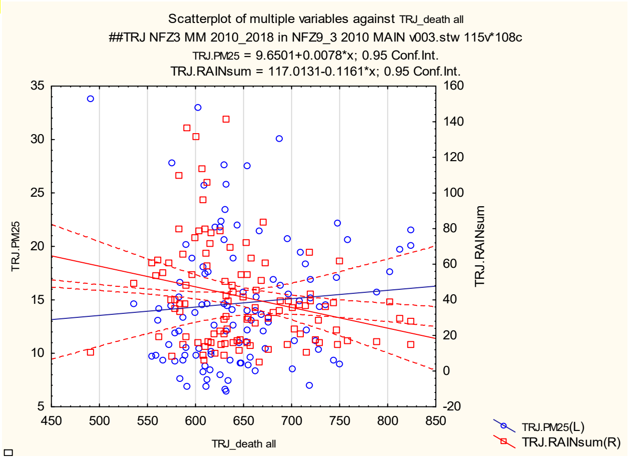

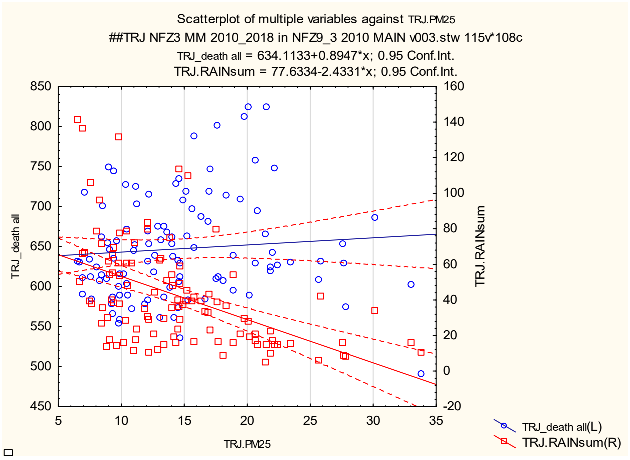

Supplementary Table 1

List of variables used for the cause-and-effect models

Variable	Description	ICD10 code	Description	ICD10 code	Description
DD	Day	TRJ_I20	Coronary artery disease	TRJ_J00	Acute inflammation of the nose and throat (common cold)
MM	Month	TRJ_I21	Acute haeart attack	TRJ_J01	Acute sinusitis
YYYY	Year	TRJ_I22	Another heart attack (reinfarction)	TRJ_J02	Acute Pharyngitis
		TRJ_I23	Some complications occurring during acute myocardial infarction	TRJ_J03	Acute tonsillitis
TRJ.SO2	SO2	TRJ_I24	Other acute forms of ischemic heart disease	TRJ_J04	Acute laryngotracheitis
TRJ.NO	NO	TRJ_I25	Chronic ischemic heart disease	TRJ_J05	Acute obstructive laryngitis and epiglottitis
TRJ.NO2	NO2	TRJ_I46	Cardiac arrest	TRJ_J06	Acute upper respiratory tract infection with multiple or unspecified localization
TRJ.NOX	NOX	TRJ_I47	Paroxysmal tachycardia	TRJ_J11	Flu caused by an unidentified virus
TRJ.O3	O3	TRJ_I48	Atrial fibrillation	TRJ_J12	Viral pneumonia, not elsewhere classified
TRJ.CO	CO	TRJ_I49	Other cardiac arrhythmia	TRJ_J13	Streptococcal pneumonia (streptococcus pneumoniae)
TRJ.CO2	CO2	TRJ_I50	Heart failure	TRJ_J14	Pneumonia caused by influenza bacillus (Haemophilus influenzae)
TRJ.PM10	PM10	TRJ_I51	Heart disease not precisely defined and complications of heart disease	TRJ_J15	Bacterial pneumonia, not elsewhere classified
TRJ.PM25	PM25	TRJ_I52	Other cardiac dysfunction in diseases classified elsewhere	TRJ_J16	Pneumonia caused by other microorganisms not elsewhere classified
TRJ.PRES	atmospheric pressure	TRJ_I63	Cerebral infarction	TRJ_J17	Pneumonia in diseases classified elsewhere
TRJ.WV	wind speed	TRJ_I64	Stroke, not defined as hemorrhagic or infarcted	TRJ_J18	Pneumonia caused by an unspecified microorganism
TRJ.TEMP	temperature	TRJ_I65	Blockage and narrowing of the pre-cerebral arteries that do not cause cerebral infarction	TRJ_J20	Acute bronchitis
TRJ.HUMID	humidity	TRJ_I66	Blockage and narrowing of the cerebral arteries that does not cause cerebral infarction	TRJ_J21	Acute bronchiolitis
TRJ.RAIN	rainfall	TRJ_I67	Other cerebrovascular diseases	TRJ_J22	Unspecified acute lower respiratory infection
TRJ.SO2.Wsea	SO2	TRJ_I68	Cerebrovascular disorders in diseases occurring elsewhere	TRJ_J30	Angioedema and allergic rhinitis
TRJ.NO.Wsea	NO	TRJ_I69	Consequences of cerebrovascular diseases	TRJ_J31	Chronic nasopharyngitis
TRJ.NO2.Wsea	NO2			TRJ_J32	Chronic sinusitis
TRJ.NOX.Wsea	NOX			TRJ_J33	Nasal polyp
TRJ.O3.Wsea	O3	TRJ_R00	Heart disorders	TRJ_J34	Other diseases of the nose and paranasal sinuses
TRJ.CO.Wsea	CO	TRJ_R05	Cough	TRJ_J35	Chronic tonsil and pharyngeal tonsil diseases
TRJ.CO2.Wsea	CO2	TRJ_R06	Breathing disorders	TRJ_J36	Peritonsillar abscess
TRJ.PM10.Wsea	PM10	TRJ_R07	Sore throat and chest	TRJ_J37	Chronic laryngitis and tracheitis
TRJ.PM25.Wsea	PM25			TRJ_J38	Inflammation of the vocal cords and larynx, not elsewhere classified
TRJ.PRES.Wsea	atmospheric pressure			TRJ_J39	Other diseases of the upper respiratory tract
TRJ.WV.Wsea	wind speed			TRJ_J40	Bronchitis not defined as acute or chronic
TRJ.TEMP.Wsea	temperature			TRJ_J41	Chronic, simple and mucopurulent, bronchitis
TRJ.HUMID.Wsea	humidity			TRJ_J42	Unspecified chronic bronchitis
TRJ.RAIN.Wsea	rainfall			TRJ_J43	Emphysema
TRJ.BaP	Benzapirene measurement results			TRJ_J44	Other chronic obstructive pulmonary disease
				TRJ_J45	Bronchial asthma
				TRJ_J46	Status asthmaticus
ShipNo	number of ships entering the port of Gdansk			TRJ_J47	Bronchiectasis
Abbreviations:					
TRJ	Tri-City Agglomeration (Gdansk)				
AMxx	designation of ARMAAG measuring stations				
Wsea	Measurement results for winds blowing from the sea				

References

1. Vignal C, Pichavant M, Alleman LY, Djouina M, Dingreville F, Perdrix E, Waxin C, Ouali Alami A, Gower-Rousseau C, Desreumaux P, et al. Effects of urban coarse particles inhalation on oxidative and inflammatory parameters in the mouse lung and colon. *Part Fibre Toxicol* (2017) **14**:46. doi:10.1186/s12989-017-0227-z

2. Xu MX, Ge CX, Li Q, Lou DS, Hu LF, Sun Y, Xiong MX, Lai LL, Zhong SY, Yi C, et al. Fisetin nanoparticles protect against PM2.5 exposure-induced neuroinflammation by down-regulation of astrocytes activation related NF-κB signaling pathway. *J Funct Foods* (2020) **65**:103716. doi:10.1016/j.jff.2019.103716

3. NHLBI. Asthma. *Natl Hear Lung Blood Inst* (2020) Available at: https://www.nhlbi.nih.gov/health-topics/asthma [Accessed November 23, 2020]

4. Mehmood T, Tianle Z, Ahmad I, Li X. Integration of AirQ+ and particulate matter mass concentration to calculate health and ecological constraints in Islamabad, Pakistan. in *16th International Bhurban Conference on Applied Sciences and Technology, IBCAST 2019* (Islamabad: Institute of Electrical and Electronics Engineers Inc.), 248–254. doi:10.1109/IBCAST.2019.8667203

5. Bebkiewicz K, Chłopek Z, Lasocki J, Szczepański K, Zimakowska-Laskowska M. The Inventory of Pollutants Hazardous to the Health of Living Organisms, Emitted by Road Transport in Poland between 1990 and 2017. *Sustainability* (2020) **12**:5387. doi:10.3390/su12135387

6. Guzdek S, Malinowski M, Religa A, Liszka D, Petryk A. Economic and ecological assessment of transport of various types of waste. *J Ecol Eng* (2020) **21**:19–26. doi:10.12911/22998993/122120

7. Benaissa F, Maesano C, Annesi-Maesano I, Alkama R. Analysis of PM concentrations in the urban area of Bejaia. *Environ Prot Eng* (2018) **44**:75–84. doi:10.5277/epe180106

8. Slama A, Śliwczyński A, Woźnica-Pyzikiewicz J, Zdrolik M, Wiśnicki B, Kubajek J, Turżańska-Wieczorek O, Studnicki M, Wierzba W, Franek E. The short-term effects of air pollution on respiratory disease hospitalizations in 5 cities in Poland: comparison of time-series and case-crossover analyses. *Environ Sci Pollut Res* (2020) **27**:24582–24590. doi:10.1007/s11356-020-08542-5

9. Mazurek H, Badyda A. *Smog: Konsekwencje zdrowotne zanieczyszczeń powietrza*. Warsaw: Państwowy Zakład Wydawnictw Lekarskich (2018).

10. Jędrak J, Konduracka E, Badyda A, Dąbrowiecki P. *Wpływ zanieczyszczeń powietrza na zdrowie*. Cracow: Krakowski Alarm Smogowy (2017).

11. Martini K, Frauenfelder T. Emphysema and lung volume reduction: The role of radiology. *J Thorac Dis* (2018) **10**:S2719–S2731. doi:10.21037/jtd.2018.05.117

12. Smith BM, Jensen D, Brosseau M, Benedetti A, Coxson HO, Bourbeau J. Impact of pulmonary emphysema on exercise capacity and its physiological determinants in chronic obstructive pulmonary disease. *Sci Rep* (2018) **8**:1–10. doi:10.1038/s41598-018-34014-5

13. Goldklang M, Stockley R. Pathophysiology of Emphysema and Implications. *Chronic Obstr Pulm Dis J COPD Found* (2016) **3**:454–458. doi:10.15326/jcopdf.3.1.2015.0175

14. Olloquequi J, Jaime S, Parra V, Cornejo-Córdova E, Valdivia G, Agustí À, Silva O. R. Comparative analysis of COPD associated with tobacco smoking, biomass smoke exposure or both. *Respir Res* (2018) **19**: doi:10.1186/s12931-018-0718-y

15. Bai JW, Chen XX, Liu S, Yu L, Xu JF. Smoking cessation affects the natural history of COPD. *Int J COPD* (2017) **12**:3323–3328. doi:10.2147/COPD.S150243

16. Korczyński P, Górska K, Jankowski P, Kosinski J, Kudas A, Sułek K, Jankowska M, Jaśkiewicz K, Krenke R. Public spirometry campaign in chronic obstructive pulmonary disease screening-hope or hype? *Adv Respir Med* (2017) **85**:143–150. doi:10.5603/ARM.2017.0024

17. NHLBI. COPD. *Natl Hear Lung Blood Inst* (2019) Available at: https://www.nhlbi.nih.gov/health-topics/copd [Accessed November 23, 2020]

18. Badyda AJ, Dąbrowiecki P, Czechowski PO, Majewski G, Doboszyńska A. Traffic-related air pollution and respiratory tract efficiency. *Adv Exp Med Biol* (2015) **834**:31–38. doi:10.1007/5584_2014_13

19. Dąbrowiecki P, Mucha D, Gayer A, Adamkiewicz Ł, Badyda AJ. “Assessment of air pollution effects on the respiratory system based on pulmonary function tests performed during spirometry days,” in *Advances in Experimental Medicine and Biology* (New York: Springer), 43–52. doi:10.1007/5584_2015_152

20. Badyda AJ, Gayer A, Czechowski PO, Majewski G, Dąbrowiecki P. Pulmonary function and incidence of selected respiratory diseases depending on the exposure to ambient PM10. *Int J Mol Sci* (2016) **17**: doi:10.3390/ijms17111954

21. Badyda AJ, Grellier J, Dąbrowiecki P. Ambient PM2.5 exposure and mortality due to lung cancer and cardiopulmonary diseases in Polish cities. *Adv Exp Med Biol* (2017) **944**:9–17. doi:10.1007/5584_2016_55

22. Jakubiak-Lasocka J, Lasocki J, Badyda AJ. The influence of particulate matter on respiratory morbidity and mortality in children and infants. *Adv Exp Med Biol* (2015) **849**:39–48. doi:10.1007/5584_2014_93

23. Rakitskii VN, Avaliani SL, Novikov SM, Shashina TA, Dodina NS, Kislitsin VA. Health risk analysis related to exposure to ambuent air contamination as a component in the strategy aimed at reducing global non-infectious epidemics. *Heal Risk Anal* (2019) **4**:1–6. doi:10.21668/health.risk/2019.4.03.eng

24. Adamkiewicz Ł, Gayer A, Mucha D, Badyda AJ, Dąbrowiecki P, Grabski P. Relative risk of lung obstruction in relation to PM10 concentration as assessed by pulmonary function tests. *Adv Exp Med Biol* (2015) **849**:83–91. doi:10.1007/5584_2014_103

25. NHS. Pneumonia. *Natl Heal Serv* (2020) Available at: https://www.nhs.uk/conditions/pneumonia/ [Accessed November 23, 2020]

26. WHO. Pneumonia. *World Heal Organ* (2020) Available at: https://www.who.int/news-room/fact-sheets/detail/pneumonia [Accessed November 23, 2020]

27. Neupane B, Jerrett M, Burnett RT, Marrie T, Arain A, Loeb M. Long-term exposure to ambient air pollution and risk of hospitalization with community-acquired pneumonia in older adults. *Am J Respir Crit Care Med* (2010) **181**:47–53. doi:10.1164/rccm.200901-0160OC
